# Oxime and thiazolidine chemoselective ligation reactions: a green method for cotton functionalization

**DOI:** 10.1007/s10570-023-05253-1

**Published:** 2023-05-18

**Authors:** Francesca Albini, Barbara Biondi, Luana Lastella, Cristina Peggion

**Affiliations:** 1 Department of Chemistry, University of Padova, 35131 Padova, Italy; 2 Institute of Biomolecular Chemistry, Padova Unit, CNR - Department of Chemistry, University of Padova, 35131 Padova, Italy

**Keywords:** Thiazolidine, Oxime, Peptide-cotton, Chemoselective ligation, Cellulose, Sustainable bioconjugation

## Abstract

During the last years, the need to create textile materials provided with peculiar properties has grown significantly. In particular, new textiles are studied to be a first protection in the prevention of living organisms from pathogens. In this regard, modifying a textile material with biologically active compounds, such as antibacterial or antiviral peptides would be useful for many applications. Our work shows a study on the possibility of modifying cotton fabrics with peptides using thiazolidine and oxime chemoselective ligations. For this purpose, an enzymatic oxidation of cellulose in a heterogeneous phase and the possibility to reuse the oxidation solution for multiple times was successfully applied. Model peptides have been designed and synthesized in order to set up the conditions for conjugating peptides to cotton via either thiazolidine or oxime bond. A systematic study of the time, pH, and quantities needed for the best reaction conditions has been conducted. The efficiency and stability of the two chemoselective ligation bonds have been studied and compared.

**Graphical abstract:**

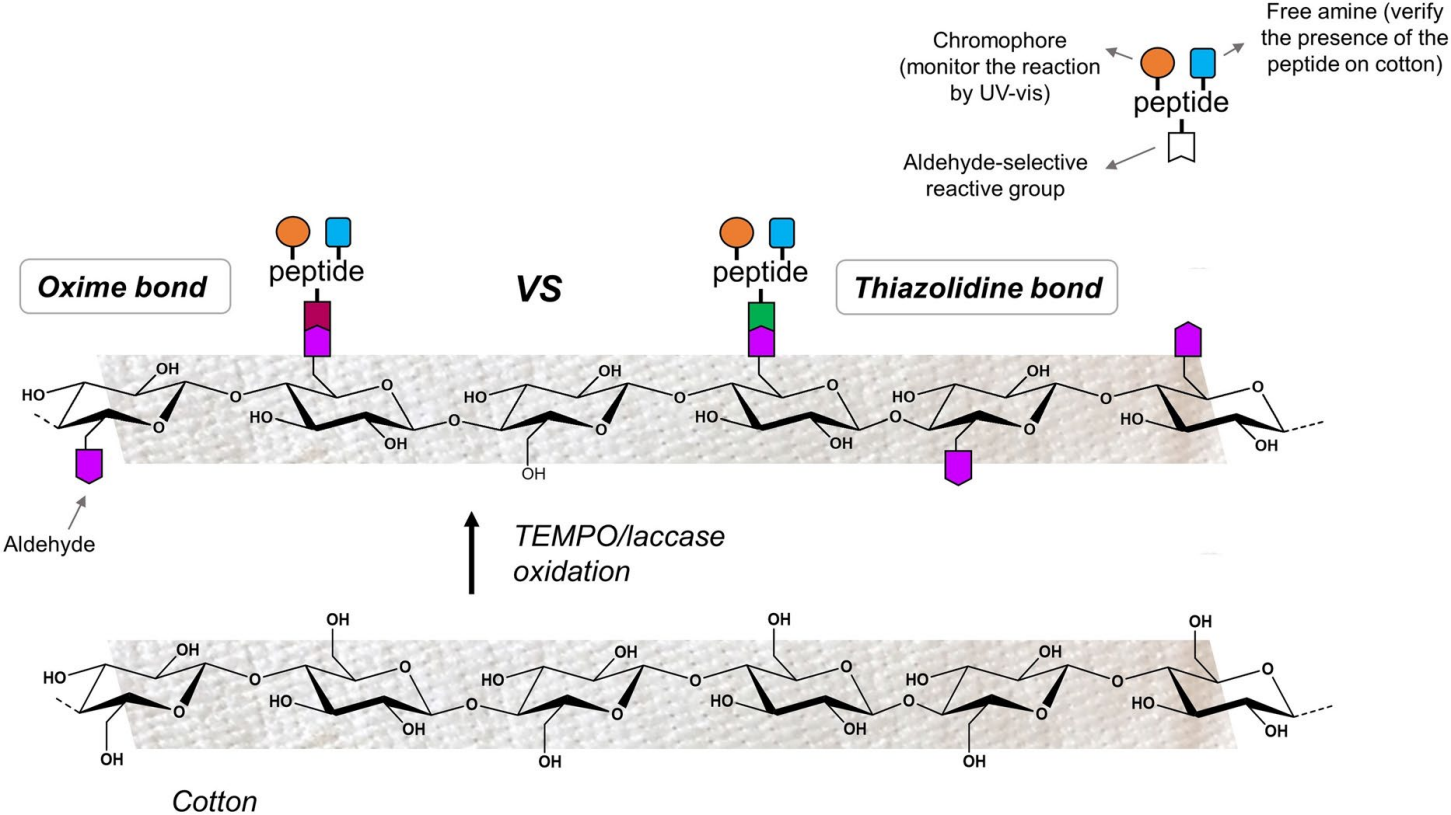

**Supplementary Information:**

The online version contains supplementary material available at 10.1007/s10570-023-05253-1.

## Introduction

In recent years, there has been growing interest in functionalized textiles for various uses ranging from biomedical to smart technology fabrics (Gomes et al. 2015; Granados et al. 2021; Grethe et al. 2015; Konwar et al. 2018; Morais et al. 2016; Zhang et al. 2016). Research is focused on the development of fabrics with biocompatible properties that are produced with sustainable technologies (Ibrahim et al. 2018; Mahmud et al. 2020; Velmurugan et al. 2020). In this context, the use of natural fibers is of great interest. In particular, cotton has several distinctive properties that make it already widely used not only in the apparel industry but also for gauze, gowns, and other medical devices. In fact, it is mainly composed of cellulose, obtainable at low cost, skin-compatible and characterized by softness, strength, elasticity, and biodegradability. However, there is a major drawback given by the hydrophilic properties of cotton that promote the growth of microorganisms (Granados et al. 2021). This phenomenon not only promotes the transmission of bacteria (Gpyal et al. 2019), but can also be the cause of unpleasant odors, degradation of fabric color, allergic reactions when the fabric comes in contact with the skin, and deterioration of the fabric itself (Zhang et al. 2016). Therefore, it would be important to find methods that can improve the bio-functional and bio-compatible properties of cotton for its safe use.

For this purpose, there is a need for molecules capable of preventing bacterial colonization of cotton fabrics, with low cytotoxicity and hopefully low propensity to develop bacterial resistance. In the search for compounds that meet the above criteria, we are helped by protein-type antibacterial agents, peptides, which have been recognized as promising candidates for surface functionalization. Peptides are naturally produced by various organisms, and therefore are naturally biocompatible and biodegradable compounds (Alves and Pereira 2014). Depending on their amino acid sequence, they present a different biological activity that spans from antimicrobial to antioxidant but also immunomodulatory (Sanchez and Vazquez 2017) and cosmeceutical (Zhang and Falla 2009). In addition, they can be easily synthetized by solid phase peptide synthesis (Amblard et al. 2006; Behrendt et al. 2015; Merrifield 1963).

Therefore, the implementation of a sustainable reaction to merge the properties of a cotton fabric with the biological activity of peptides into a single material would be quite significant. To ensure that the functionalized fabric maintains the acquired properties over time, an efficient method of conjugation is the use of a covalent bond. In this context, the concept of chemoselective ligation is particularly interesting. It was first introduced to synthesize long-chain peptides from shorter amino acid chains that contained unique and mutually reactive functional groups capable of ligation by a one-pot reaction (Kent and Schnolzer 1992). The reactions are so selective that they do not involve other reactive groups in the peptide side chains. Nowadays it is widely exploited for the total chemical synthesis of proteins (Kent 2009), to introduce new structural features into the protein or nanoparticles structure, or to functionalize biomaterials (Ramakers et al. 2014).

Of particular significance are the thiazolidine and oxime bonds. The first is formed after the reaction between an aldehyde and a β-amino thiol, usually the side chain of cysteine (Liu and Tam 1994; Tam et al. 1994) and was tested by Scapin et al. to bind an antimicrobial peptide on a cotton textile (Scapin et al. 2020). The second is obtained by the reaction between a carbonyl moiety and a α-nucleophile of an aminooxy acetic acid (Kolmel and Kool 2017). In both cases, a slightly acidic pH is ideal for the conjugation, the reaction takes place at room temperature, and the only by-product is water (Shao and Tam 1995).

To obtain thiazolidine and oxime bonds, an aldehyde moiety is required on cotton, which is achievable by oxidation of the cellulose hydroxyl groups. Among the methods reported (Aracri et al. 2012; Cumpstey 2013; Pierre et al. 2017; Potthast et al. 2007, 2009) periodate oxidation is one of the most widely used for polysaccharides (Nypelö et al. 2021). This type of oxidation takes place at the C2-OH and C3-OH positions and leads to ring scission, altering the structure of the polysaccharide chain. It is often reported that this oxidation method increases flexibility of the polymeric backbone but also causes a degradation of the cellulose chain (Errokh et al. 2018; Potthast et al. 2007, 2009; Sun et al. 2015) Furthermore, it was demonstrated that on yarn cotton fabrics periodate oxidation alters the mechanical properties of the fiber itself, resulting in a damaged material (Scapin et al. 2020). For this reason, within this framework we choose the laccase chemoenzymatic oxidation mediated by 2,2,6,6- tetramethylpiperidine-1-oxyl (TEMPO), as an alternative milder oxidation method, largely selective for C6-OH position. Indeed, in this reaction, the primary alcohols of cellulose are selectively oxidized to aldehydes under mild reaction conditions without altering structural integrity of the polysaccaride (Aracri et al. 2012; Coseri et al. 2013; Tromp et al. 2010; Viikari et al. 2000).

This paper reports the study of two chemoselective reactions for the functionalization of oxidized cotton with peptides. This was done by designing and synthesizing two model peptides: H-Cys-Gly-Trp-Lys-NH_2_ (peptide **a**) for thiazolidine bond formation and H-Aox-Gly-Trp-Lys-NH_2_ (peptide **b**) for oxime bond formation. Both oxidation and conjugation reactions herein presented occur in an aqueous solvent under mild conditions. Different reaction conditions were explored, and the two covalent bonds were compared and optimized.

## Methods and materials

### Reagents and solvents

Protected amino acids, Fmoc Rink Amide AM resin, and coupling reagents were purchased from Iris Biotech. All other chemicals were Sigma-Aldrich products and were used without further purification. DMF was always previously fluxed with nitrogen when used in the synthesis of peptides. Cotton samples were supplied by Santex Spa (medical gauze cotton, G-cotton) and Piave Maitex Srl (organic cotton for textiles, T-cotton). The T-cotton did not require further treatments prior to use, while the G-cotton needed to be mercerized prior to use.

### Mercerization of cotton

G-cotton was stirred in a 1% NaOH solution heated to reflux. When the solution became yellow, it was replaced by a fresh NaOH solution. This process was continued until the color change was no longer detectable. The cotton was then washed with water and MeOH and finally dried in a desiccator.

### Oxidation of cotton samples

The oxidation solution was prepared by dissolving 20 U/g_cotton_ of laccase (laccase from *Trametes Versicolor*; 0.89 U/mg from Sigma-Aldrich) and 8% (m/m_cotton_) of 2,2,6,6- tetra methylpiperidine-1-oxyl (TEMPO) in 30 mL/g_cotton_ of acetate buffer (50 mM, pH = 5) with a procedure similar to that described by Aracri et al. (Aracri et al. 2012). Cotton samples (3 g) were stirred in the oxidation solution for 2 days at room temperature. The oxidized cottons were then washed with acetate buffer (20 mL × 3), water (20 mL × 5) and acetone (20 mL × 5). Cotton samples were dried in a desiccator under reduced pressure for at least one night. Both G- and T-cotton were subjected to this procedure.

The presence of aldehydes was qualitatively assessed on the oxidized cottons by soaking a piece (about 0.2 × 0.2 cm) or a thread (about 0.5 cm) of cotton in the Schiff’s reagent solution for 10 min (Robins et al. 1980) (commercially available solution from Sigma-Aldrich). The color was then compared with that of an unoxidized cotton subjected to the same procedure.

### Second and third use of the oxidation solution

A piece of G-cotton was added to 30 mL/g_cotton_ of the laccase/TEMPO solution used for the previous oxidation and stirred for 4 days at room temperature. The presence of the aldehydes was evaluated by a *Schiff test*. Then, the oxidized cotton was washed with acetate buffer (20 mL × 3), water (20 mL × 5) and acetone (20 mL × 5). Cotton samples were dried in a desiccator under reduced pressure. This procedure was performed twice on G-cotton.

From this point on the oxidized cotton derived from the first, second and third reuse of the peptide solution will be referred to as G1- or T1-cotton, G2-cotton, and G3-cotton, respectively (unless otherwise specified).

### Synthesis of peptides and characterization

Both peptides (Table 1) were synthetized on a 0.3 mmol scale by manual solid phase peptide synthesis using filter-equipped syringes. For peptide **a**, Fmoc Rink Amide AM resin (500 mg, 0.65 mmol/g loading) was swelled in DMF (1 h). Then, Fmoc-Lys(Boc)-OH, Fmoc-Trp(Boc)-OH, Fmoc-Gly-OH, and Fmoc-Cys(Trt)-OH were added step-by-step using the Fmoc strategy. At each step 3 Eq. (0.9 mmol) of protected amino acid were activated with 3 eq. of Oxima/DIC using DMF as the solvent (1 h). Deprotection of the Fmoc group was carried out with 20% piperidine solution in DMF (1 × 5 min + 1 × 15 min). The removal of the peptide from the resin was achieved with a solution with a 94/2.5/2.5/1 ratio of TFA/DODT/H_2_O/TIS (2 h).

**Table 1:** List of the synthetized model peptides

Peptide	Sequence
a	H-Cys-Gly-Trp-Lys-NH_2_
b	H-Aox-Gly-Trp-Lys-NH_2_

For peptide **b,** Fmoc Rink Amide AM resin (500 mg, loading 0.65 mmol/g) was swelled in DMF (1 h). Then, Fmoc-Lys(Boc)-OH, Fmoc-Trp(Boc)-OH, Fmoc-Gly-OH, and Boc-Aox-OH were added step by step using the Fmoc strategy. At each step 3 Eq. (0.9 mmol) of protected amino acid were activated with 3 eq. of Oxima/DIC using DMF as the solvent (1 h). Deprotection of the Fmoc group was carried out with 20% piperidine solution in DMF (1 × 5 min + 1 × 15 min). The removal of the peptide from the resin was achieved with a solution with a 95/2.5/2.5 ratio of TFA/H_2_O/TIS (2 h). After cleavage of the resin, the peptides were precipitated with Et_2_O, the solution was centrifuged (10 min at 6000 rpm), and the supernatant was removed. The peptides were then dried in the desiccator under reduced pressure.

The peptides were analyzed with a Phenomenex C_18_ reverse phase column (4.6 × 250 mm, 5 μ, 100 Å) using a VWR HITACHI Chromaster instrument. The binary elution system used was A, 0.05% TFA in CH_3_CN/H_2_O (1:9 v/v); B, 0.05% TFA in CH_3_CN/H_2_O (9:1 v/v); and spectrophotometric detection at λ = 214 nm and λ = 280 nm. ESI–MS was performed using an Agilent technologies 1260 Infinity II instrument equipped with an Agilent technologies quadrupole LC–MS 6130. The amino acid sequence, mass spectral data, and HPLC retention time of the two peptides are as follows:

[H-Cys-Gly-Trp-Lys-NH_2_] (peptide **a**) HPLC: Rt = 6.49 min (0–30% B in 30 min); mass: calculated 491.61; found 492.2 [M + H]^+^. [H-Aox-Gly-Trp-Lys-NH_2_] (peptide **b**) HPLC: Rt = 6.89 min (0–30% B in 30 min); mass: calculated 461.24; found 462.3 [M + H]^+^.

The peptides chain sequence was also confirmed by NMR (Supporting Information).

### Peptide-cotton conjugation

A weighted piece of oxidized cotton was dipped into a solution of the chosen peptide, dissolved in 50 mM acetate buffer at pH 5. In the case of peptide **a**, 1 eq. of Tris(2-carboxyethyl)phosphine (TCEP) was added. The reaction was monitored by UV-vis following the absorbance of Trp (at 280 nm) in solution. A Shimadzu UV-vis 250 1PC with a 350–230 nm interval, 2 nm slit, and a 0.1 nm sampling interval was used. Depending on the experiment, quartz Hellma Analytics cuvettes with an optical path of 1 cm or 1 mm were used.

The peptide solution was stirred at room temperature until the decrease in absorbance stopped. Then, the cotton piece was removed from the solution, washed with water (3 mL × 7) and acetone (3 mL × 7) and dried in a desiccator. The presence of the peptides on cotton was assessed by a *Kaiser test* (Kaiser et al. 1970) and the absence of free aldehyde sites was checked with the *Schiff’s reagent.* Variable conditions of peptide concentration and peptide excess (with respect to cotton) were investigated (Table 2). Unoxidized T-cotton was subjected to the same procedure as a negative control.

**Table 2:** Operating conditions used for the peptide-cotton conjugations

		Reaction	Description		Peptide-cotton Ratio (mmol/g_cotton_)		Peptide concentration (mM)		Peptide Loading (mmol/g_cotton_)		Reaction time
Thiazolidine reactions		**r1**	G1-cotton + **a**		0.44		0.17		0.16		3 days
		**r2**	T1-cotton + **a**		0.63		0.17		0.15		3 days
		**r3**	G1-cotton + **a**		0.99		2.2		0.16		1 day
		**r4**	T1-cotton + **a**		1.2		1.1		0.10		1 day
		**r5**	T1-cotton + **a** (a reused from **r4**)		2.0		1.0		0.10		1 day
		**r6**	T1-cotton + **a** (a reused from **r5**)		1.9		0.91		0.11		1 day
		**r7**	G1-cotton + **a**		1.4		1.1*		0.15		1 day
			G2-cotton + **a**		1.5				0.12		
			G3-cotton + **a**		1.3				0.07		
		**r11**	T-cotton + **a**		0.80		0.72*		0.08		1 day
			T1-cotton + **a**		0.69				0.01		
Oxime reactions		**r8**	T1-cotton + **b**		2.0		1.7		0.13		1 day
		**r9**	T1- cotton + **b** (**b** reused from **r8**)		1.90		1.59		0.09		1 day
		**r10**	T1-cotton + **b** (**b** reused from **r9**)		2.16		1.50		0.09		1 day
		**r12**	T-cotton + **b**		0.73		0.77*		0.09		1 day
			T1-cotton + **b**		0.76				0.004		

Solutions of peptide **a** and peptide **b** are simply referred to as **a** and **b**.^*^ The same solution was split in two/three reaction vessels and used for the two/three experiments

The peptide loading on cotton is intended as the millimoles of peptide linked to cotton and was calculated as follows:

1 $$\frac{{\frac{{A_{i} - A_{f} }}{\varepsilon \cdot l} \cdot V{ }\left[ {mmol} \right]}}{{m_{cotton} { }\left[ g \right]}}$$

where A_f_ and A_i_ are the final and initial absorbance of the peptide solution measured at 280 nm (maximum of Trp), respectively. ε is the extinction coefficient of Trp at 280 nm (5630 M^−1^ cm^−1^) (Pace et al. 1995), l is the optical path of the cuvette and V the volume of the peptide solution.

### Characterization of the peptide-cotton conjugates with FT-IR

A Nicolet nexus 670 spectrophotometer was used to collect the FT-IR spectra. The cotton samples were mechanically frayed and incorporated in a KBr pellet. The chamber where the samples sit was maintained under a constant N_2_ flow to minimize the contributions of H_2_O and CO_2_. The spectra were collected through 20 scans in the range 4000–400 cm^−1^, with a resolution of 2 cm^−1^. Samples of G-and T-Cotton, G- and T- oxidized cotton and the functionalized cottons were subjected to this measurement.

### Characterization of the peptide-cotton conjugates with XPS

X-ray photoelectron spectra (XPS) were recorded on an Escalab QXi multi-technique surface analysis instrument (Thermo scientific) with a standard Al Kα monochromatic source and electrostatic lens; charge compensation was obtained with flood gun. Pass energy was selected at 200 eV for survey spectra and 50 eV for single regions (Al 2p, Ce 3d, Cu 2p, Fe 2p, O 1 s); channel size was 0.5 eV (50 ms/channel) for survey spectra and 0.1 eV (100 ms/channel) for single regions. Instrument calibration was carried out using Au 4f peak. The quantitative analysis was carried out after Shirley-type background subtraction. For qualitative analysis NIST X-ray Photoelectron Spectroscopy Database (Database 20, Version 4.1 https://doi.org/10.18434/T4T88K) was used in addition to specific papers. Fitting was carried out using Voight function and Instrumental “Smart” background. T- oxidized cotton and the functionalized cottons from reactions **r4** and **r9** were subjected to this measurement (Supporting Information).

### Stability of the peptide-cotton bonds

The release of peptides **a** and **b** from B-cotton (from reactions **r4** and **r8**) was investigated at pH 3 (50 mM sodium citrate/citric acid buffer), pH 7 (PBS buffer) and pH 10 (50 mM sodium carbonate/sodium bicarbonate buffer), in HCl 3 M and in 70%_v/v_ EtOH at 30 °C. For each experiment, 2.5 mL of the chosen solution was placed in a cuvette together with the functionalized cotton (1 cm optical path). The solutions were stirred for 3 days and its absorbance at 280 nm was checked at 0 min, 2, 4, 6 h, 1, 2 and 3 days.

The percentage of peptide loss was calculated as follows:

2 $$\frac{{\frac{A}{\varepsilon \cdot l} \cdot V}}{{n_{bound} }} \cdot 100$$

where A is the absorbance of the peptide solution measured at 280 nm at a given timepoint, ε is the extinction coefficient of Trp at 280 nm (5630 M^−1^ cm^−1^) (Pace et al. 1995), l is the optical path of the cuvette and V the volume of the cuvette used. The moles of the peptide bound to cotton are easily calculated knowing the peptide loading and the mass of cotton used.

After the reactions took place, the cottons were removed from the solutions, washed with water (3 mL × 7) and acetone (3 mL × 7) and dried in a drier. The cottons were then subjected to *Kaiser* and *Schiff* tests.

## Results and discussion

### Peptide design

The model peptides H-Cys-Gly-Trp-Lys-NH_2_ (**a**) and H-Aox-Gly-Trp-Lys-NH_2_ (**b**) were selected to study the thiazolidine and oxime bond on oxidized cotton, respectively (Table 1).

**Fig. 1:**
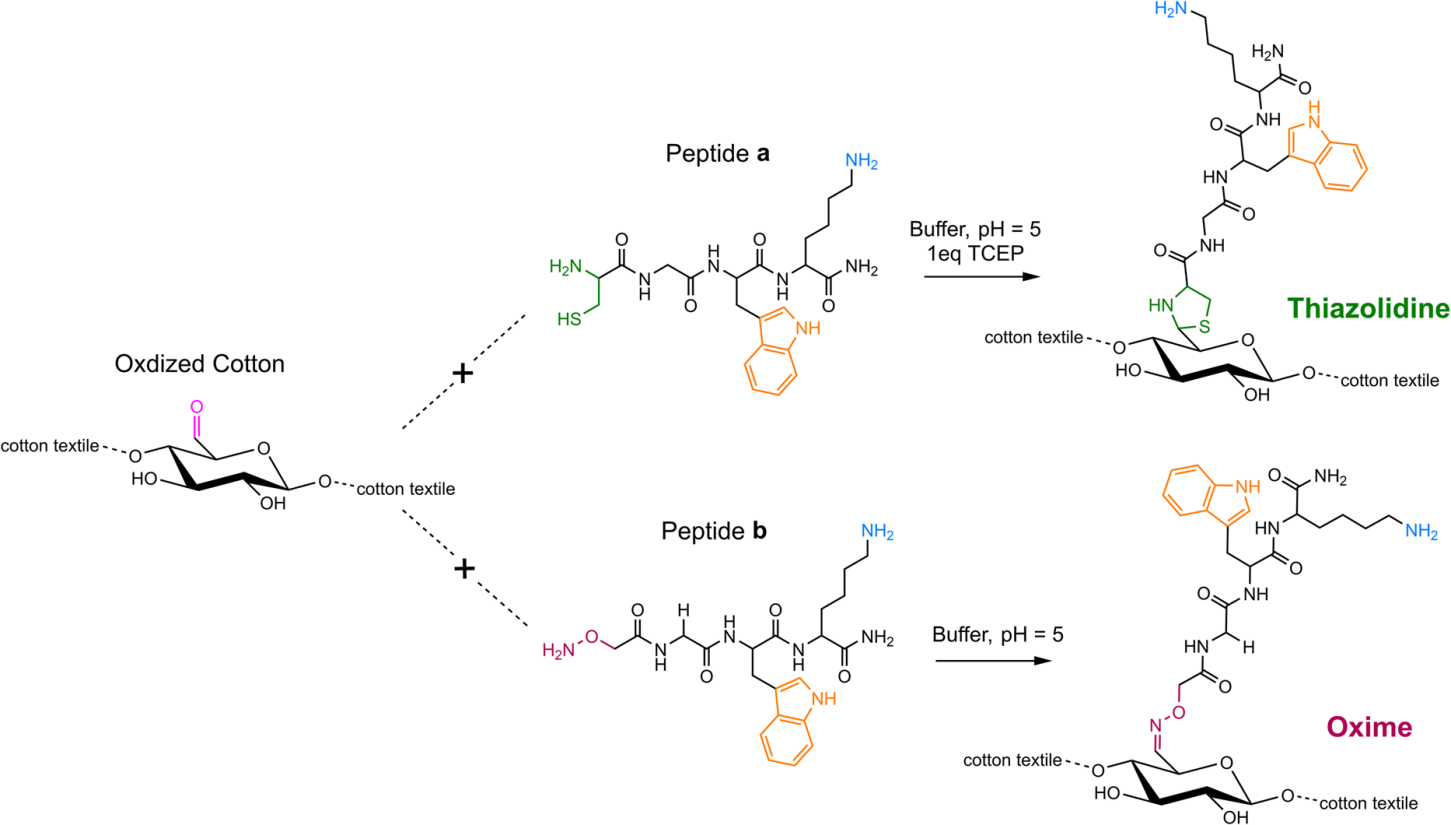
Thiazolidine and oxime bond formation between oxidized cotton and H-Cys-Gly-Trp-Lys-NH_2_ (**a**) or H-Aox-Gly-Trp-Lys-NH_2_ (**b**). The aldehyde (pink), the chromophore (orange), and the primary amine (blue) are highlighted

The amino acid chains were designed to contain the functional groups necessary to achieve thiazolidine and oxime bonds. The amino acids in the sequence were chosen so that they contained the chromophores and functionalities necessary to monitor the cotton-conjugation reactions and the stability of the obtained bonds. An aldehyde-selective reactive group (Cys or Aox) was inserted at the N-terminus. In fact, the thiazolidine bond is formed between the aldehydes present on the cellulosic chain of oxidized cotton and the N-terminal Cys of peptide **a**, while to form the oxime bond the aldehydes react with the N-terminal Aox of peptide **b** (Fig. 1).

The presence of Trp provided the peptides with a chromophore that allowed the reaction to be followed by UV–vis, and thus the amount of peptide on cotton could be estimated. Additionally, the free amine on the Lys side chain makes it easy to determine the actual presence of the peptide on cotton by a *Kaiser test*.

### Oxidation of cotton

The laccase/TEMPO catalysis system (Aracri et al. 2012; Tromp et al. 2010; Viikari et al. 2000) described by Aracri et al. (Aracri et al. 2012) was often employed to generate aldehydes on cellulose. The use of the laccase enzyme provides a halide-free method to regenerate reduced TEMPO using O_2_ as primary oxidant (Tromp et al. 2010). Laccase-assisted TEMPO oxidation allows in-situ regeneration of the nitrosonium salt, where only oxygen is the final electron acceptor during the reaction (Pierre et al. 2017).

TEMPO preferentially oxidizes C6-OH to aldehyde, with a selectivity (> 95%) that is high enough not to alter the cellulose chain backbone (Coseri et al. 2013; Pierre et al. 2017). This degree of selectivity ensures that at the end of the oxidation reactions the cotton structural integrity is maintained. Furthermore, this enzymatic reaction that takes place in an aqueous solution at slightly acidic pH and room temperature, assuring mild reaction conditions that do not damage yarned cotton.

In this work, the reaction was conducted on cotton in a heterogeneous system due to the insolubility of cotton itself. Two types of cotton were used, a wide-weave medical gauze called G-cotton and a thicker-weave organic cotton fabric called T-cotton. The G-cotton was only washed with water before use, while the G-cotton was mercerized by refluxing into a NaOH solution until the removal of its distinctive yellow coloration.

Thus, to introduce aldehyde functionalities on both cotton the procedure illustrated by Aracri et al. was followed. Both G-cotton and T-cotton, were kept under stirring in a 20U/g_cotton_ of laccase/8% m/m_cotton_ TEMPO solution for two days. This oxidation mixture was chosen to maximize the amount of formed aldehydes (Aracri et al. 2012). In our experiment, air oxygen supplied by stirring was sufficient for the reaction to undergo.

The Schiff reagent was used qualitatively to assess the success of the oxidation reaction by comparing the coloring of oxidized and unoxidized cotton (Robins et al. 1980). When unoxidized cotton is subjected to Schiff test, no visible change in color is observed, indicating a poor and/or undetectable reactivity of the reducing aldehydes at the end of the cellulose chains. In contrast, oxidized cotton under the same conditions assumes a pink color, which is indicative of the formation of additional aldehydes.

Recycling and recovery of reagents is an issue of utmost importance in oxidation reactions of potential industrial interest. In this context TEMPO/laccase oxidation system could be a good candidate for optimising green chemical synthesis, as the the only by-product of the reaction is water. In this context, we investigated the possibility of reusing the oxidation solution multiple times.

**Fig. 2:**
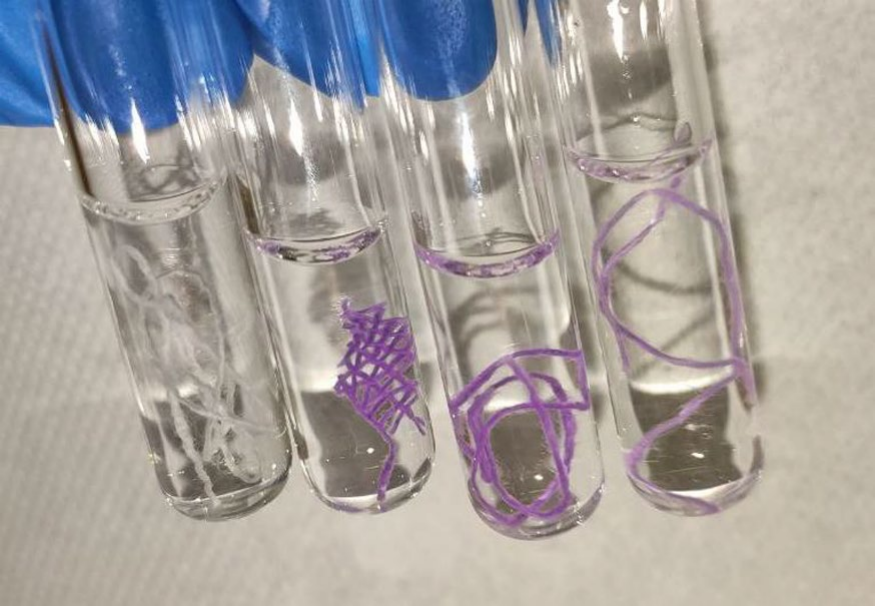
From the left, *Schiff test* on G-cotton, oxidized G-cotton from the 1st use of the oxidation solution, oxidized G-cotton from the 2nd use of the oxidation solution, oxidized G-cotton from the 3rd use of the oxidation solution

To this aim, after the removal of the first piece of oxidized cotton (G1-cotton), a second piece of cotton (G2-cotton) was added to the TEMPO/laccase solution and stirred for four days. And after the second was removed, a third piece of cotton (G3-cotton) was added and stirred for four days. Reuse of the solution required longer times of reaction, but aldehyde formation was still successful. However, the lower color intensity of the *Schiff test* on G3-cotton (Fig. 2) suggests a diminished degree of oxidation upon multiple oxidations.

We were unable to quantify this decrease by UV–vis as, in this application, the chromophore formed between the aldehydes and the Schiff reagent remains attached to cotton.

These observations were supported by the results obtained from the reaction of G1-cotton, G2-cotton and G3-cotton with peptide **a** (Table 2, reaction **r7**). In fact, the three pieces of cotton were functionalized simultaneously using the same peptide solution placed in three different reaction vessels. The peptide loading obtained decreases from cotton obtained with the fresh oxidizing solution to those obtained with successive reuses of the solution (Table 2). This can be attributed to a lower amount of aldehyde functions on the cotton pieces.

With these experiments we demonstrated that the oxidizing solution can be used at least up to three times, although the oxidizing power decreases in subsequent reuses. Additionally, the fact that the reaction takes place in the heterogeneous phase is of practical interest for a large-scale application. In practice, the yarned cotton can be dipped in the oxidising solution and simply removed, without quenching the solution that can then immediately be used to oxidize another cotton sample.

### Peptide-cotton conjugation by thiazolidine

The formation of the thiazolidine ring between peptide **a**, Cys of H-Cys-Gly-Trp-Lys-NH_2_, and the aldehydes present on the oxidized cottons was easily achieved with a mild reaction in aqueous buffer at pH 5. The reaction needs the presence of a reducing agent, TCEP, in solution to avoid the formation of dimers consequent to disulfide bridge formation between thiol groups of Cys. With a concentration higher than 1 mM the reaction was completed in only one day, whereas peptide concentration of 0.1 mM requires reaction times of at least three days (Table 2).

Peptide-cotton bond formation was monitored by UV absorption, using the Trp probe inserted into the peptide sequence for this purpose. As the reaction proceeds, the Trp absorption signal decreases, indicating that the peptide is being subtracted from the solution as it binds to the cotton.

**Fig. 3:**
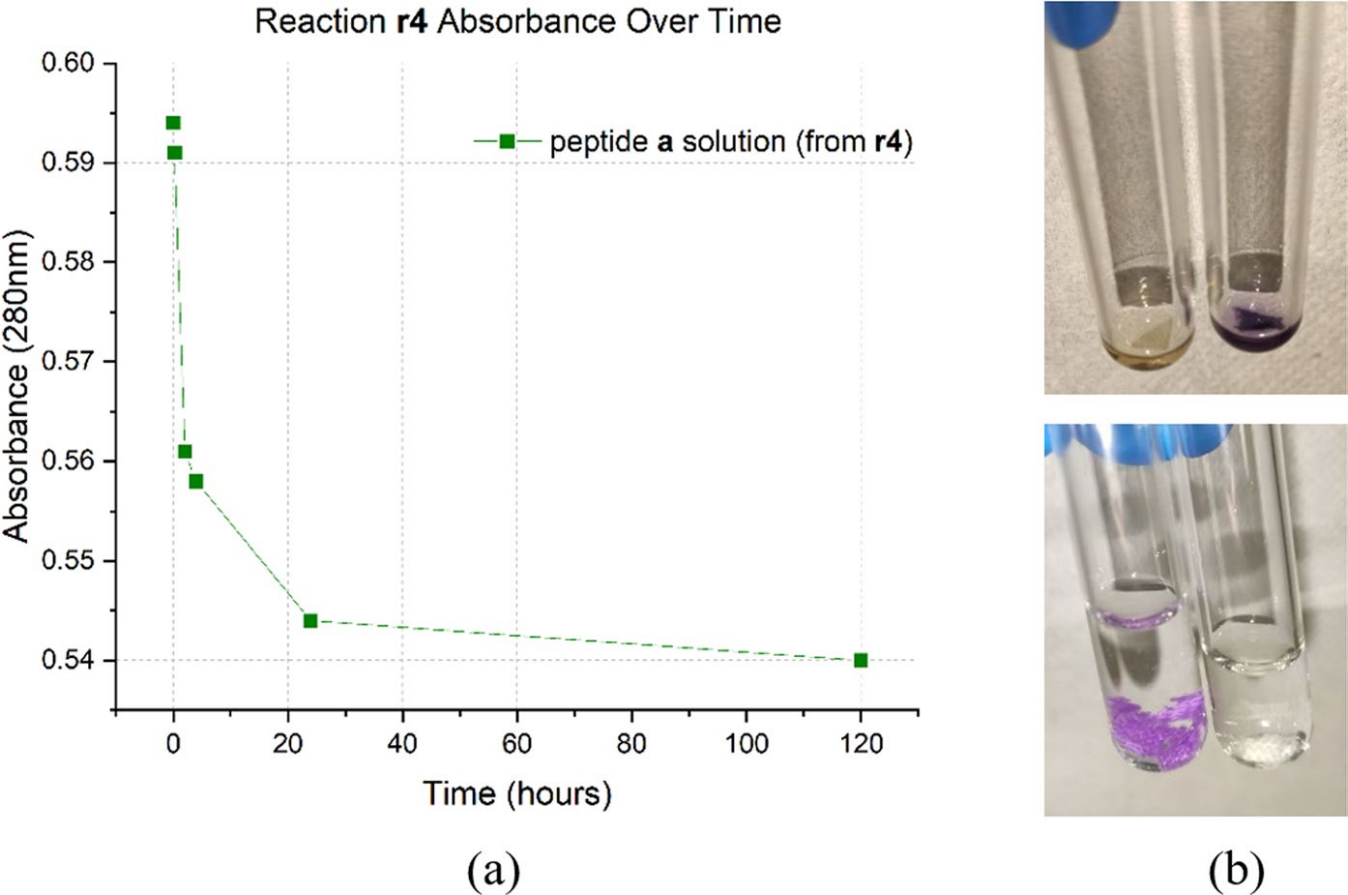
**a** Absorbance at 280 nm over time of peptide** a** solution in reaction **r4**. **b** Kaiser test (top) and Schiff tests (bottom): on the left an oxidized T-cotton sample used as control, on the right the functionalized T-cotton from **r4**

Figure 3 is shown as an example and represents the absorbance at 280 nm as reaction **r4** is performed. The absorbance of the Trp at 280 nm decreases over time, confirming the subtraction of peptide **a** from the solution. This allowed the calculation of the difference in peptide concentration at selected times through Lambert–Beer’s law and thus the amount of peptide linked to cotton, expressed as peptide loading.

The difference in the weave of G- and T-cotton did not result in drastic differences in the peptide loading or the reaction time needed to link peptides (Table 2, reactions **r1** and **r2**; **r3, r4** and **r7**). However, the use of T-cotton was generally preferred because of the tendency of G-cotton to disintegrate over the reaction time.

The presence of peptide **a** on cotton was confirmed by the *Kaiser test*, which resulted blue for each piece of functionalized cotton. In addition, the *Schiff test* performed on the functionalized cotton pieces did not show any pink staining, suggesting a quantitative reaction with the aldehyde sites.

To maximize the functionalization of cotton, the peptide has always been used in large excess over reactive sites on cotton. Therefore, the possibility of reusing the peptide solution for further reactions was considered. To do this, in reaction **r5** a known volume of the solution previously used in reaction **r4** (Table 2) was used to functionalize another piece of cotton. Before reuse, the solution was filtered to remove all possible cotton residues from the previous conjugation, and 1 eq. of TCEP was added to prevent the formation of peptide **a**—peptide **a** dimers. In reaction **r6** the same procedure was performed, using the peptide solution of reaction **r5**. Interestingly, the results obtained in reactions **r5** and **r6** did not show significant differences, in terms of peptide loading or reaction time, compared to reaction **r4**.

### Peptide-cotton conjugation by oxime

Substitution of N-terminal Cys with aminooxyacetic acid, Aox, in the model peptide **b** sequence was studied to evaluate whether oxime linkage could be a better alternative to thiazolidine. Indeed, Aox reacts with aldehydes under the same reaction conditions as Cys, but during the conjugation reaction there is no risk of dimerization of the peptide. In fact, in the absence of SH groups, the peptide solution can be concentrated sufficiently to facilitate the formation of the new bond. The peptide can thus be used in large excess with respect to the aldehyde sites to be reacted, without the need to dilute the reaction solution, nor to add the reducing agent TCEP.

Therefore, the best reaction conditions developed in the study of the thiazolidine forming reaction were used as a starting point for setting up the oxime forming reaction. In particular, because the concentration of the peptide solution plays an important role in the success of the conjugation reaction, the peptide concentration was always kept at about 1 mM and peptide **b**, H-Aox-Gly-Trp-Lys-NH2, was used in large excess.

The results, summarized in Table 2, show that a peptide loading of approximately 0.1 mmol/g_cotton_ was obtained in a reaction time of one day. This indicates that both Cys of peptide **a** and Aox of peptide **b** reacted with most of the aldehydes on the cotton surface, thus saturating the cotton sites. Again, the success of the reaction was confirmed by the *Kaiser* and *Schiff tests*.

**Fig. 4:**
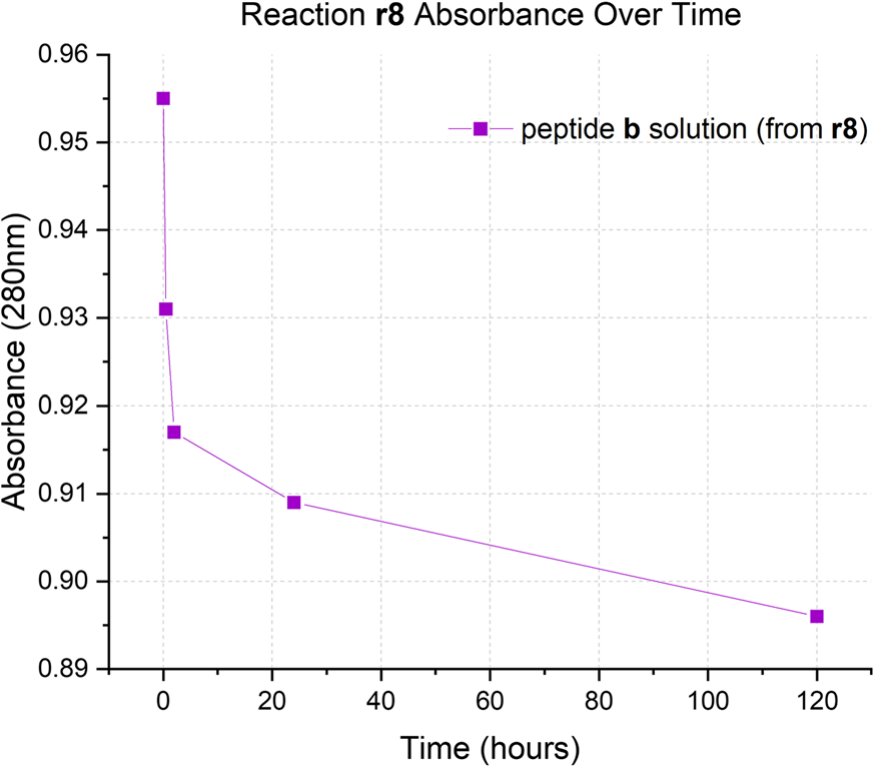
Absorbance at 280 nm over time of peptide **b** solutions in reaction **r8**

**Fig. 5:**
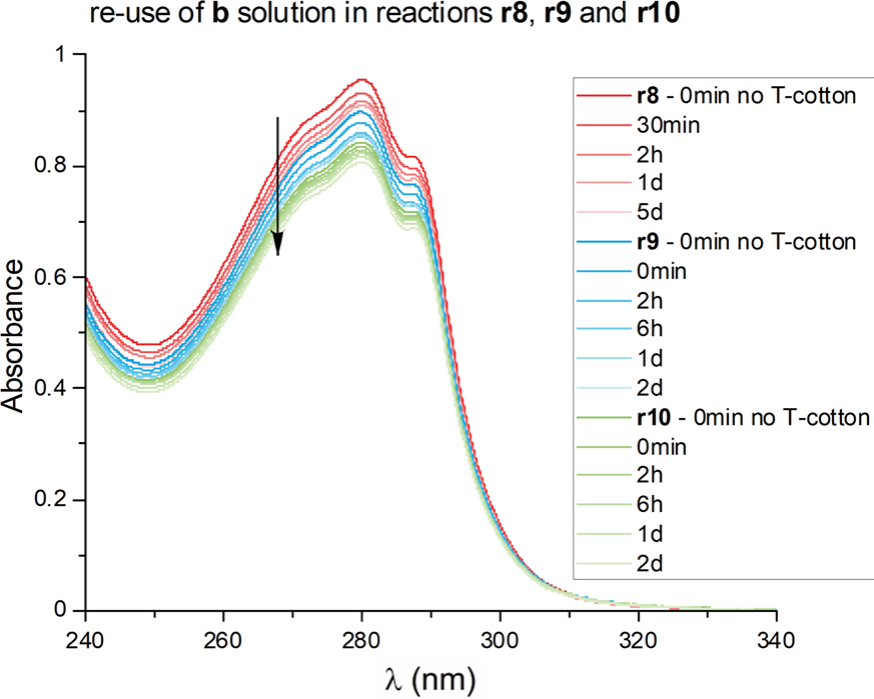
UV–vis spectra of reactions **r8** (red), **r9** (blue), and **r10** (green)

Similarly to the experiments carried out with peptide **a**, the solution of peptide **b** was also re-used twice without observing a change in peptide loading, nor the need to lengthen the reaction time (Fig. 5). In fact, data from reactions **r9** and **r10** show a loading value of 0.09 mmol/g, only slightly lower than the loading obtained with the first use of the solution (experiment **r8**, Fig. 4) in which the loading obtained was 0.13 mmol/g (Table 2). All reactions have a duration of 1 day.

To ensure that the peptides were covalently bound to the cellulose matrix by thiazolidine or oxime ligations and not just absorbed into the fiber, oxidized and unoxidized cotton were added to the same peptide solution that was split into two reaction vessels (**r11** and **r12**). The absorbance of both peptide solutions did not decrease significantly over time when unoxidized cotton was present (Fig. S4 in Supporting Information). In contrast, the behavior in the presence of oxidized T1-cotton was similar to previous experiments.

### FT-IR and XPS characterization of the conjugate

In this study, the FT-IR spectra of cotton, oxidized cotton, and cotton functionalized with peptide **a** were compared. The spectra of G- and T-cotton are consistent with data in the literature (Vaideki et al. 2007) and the two types of cotton do not differ significantly from each other (Supporting Information). In the C=O stretching zone, the signals of the aldehyde group expected for oxidized cotton, are not visible at 1740 cm^−1^ probably because aldehydes are masked by their hemiacetal and hydrated forms (Amer et al. 2016; Kim et al. 2000), slightly detectable as a broad band in the 880 cm^−1^ region.

**Fig. 6:**
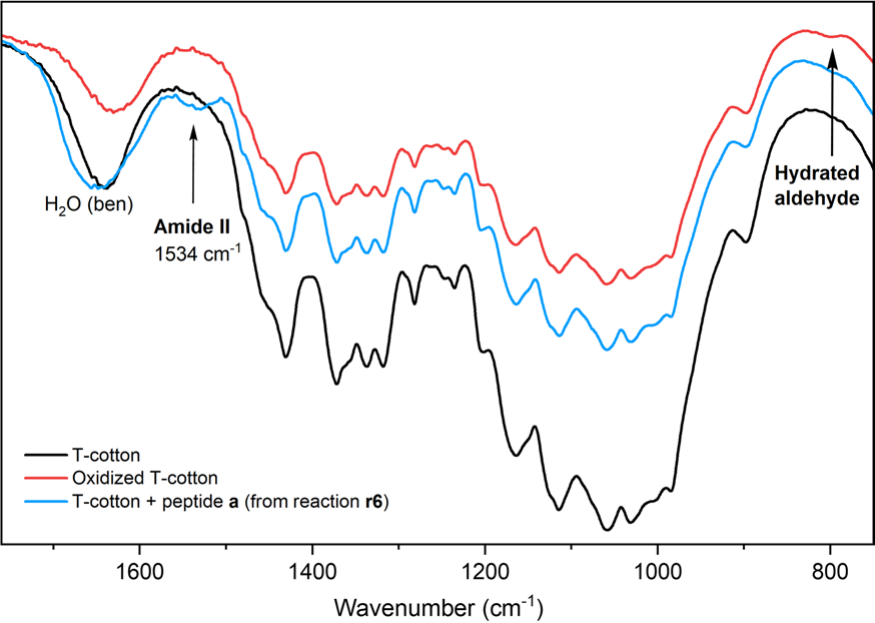
FT-IR spectra (KBr disc) of T-cotton (black), oxidized T1-cotton (red) and T1-cotton functionalized with **a** from **r6** (blue) recorded in the 1750–1250 cm^−1^ region

However, it was possible to detect the presence of the peptide on cotton through the Amide II band (C-N stretching and N–H bending) (Long 2004) at 1534 cm^−1^. Although relatively weak, this characteristic band is present in all the spectra of functionalized cottons. As a representative example, Fig. 6 shows the FT-IR of T-cotton, oxidized T-cotton, and T-cotton functionalized with conjugation reaction **r6**.

We carried out a XPS (X-ray Photoelectron Spectroscopy) analysis to evaluate the atomic composition of the peptide functionalized cotton. XPS analysis confirmed the presence of both peptides on functionalized pieces of cotton. In fact, nitrogen atoms are not supposed to be present in the cellulose matrix, but are expected when the peptide is conjugated. Indeed, as shown in Table 3, nitrogen was detectable only in the samples functionalized with either peptide **a** or **b**.

**Table 3:** Atomic concentration of C, N and O detected by X-ray Photoelectron Spectroscopy (XPS) on oxidized T-cotton and T-cotton functionalized with peptide **a** and **b**, respectively from reaction **r4** and **r9**

Reaction	Description	%C	%N	%O
–	T1-cotton	19.34	–	80.42
r4	T1-cotton + peptide **a**	64.33	2.72	32.95
r9	T1-cotton + peptide **b**	63.87	2.30	33.82

### Stability of the cotton-peptide bonds

Due to their inherently stability, oximes and thiazolidines are typically used in bioconjugation reactions, and, together with hydrazones, they are employed for the controlled release of biologically active molecules. The higher stability, compared to that of imines, is ascribable mainly to the negative inductive effect of the heteroatom (O or S) (Bermejo-Velasco et al. 2018; Kolmel et al. 2017).

**Fig. 7:**
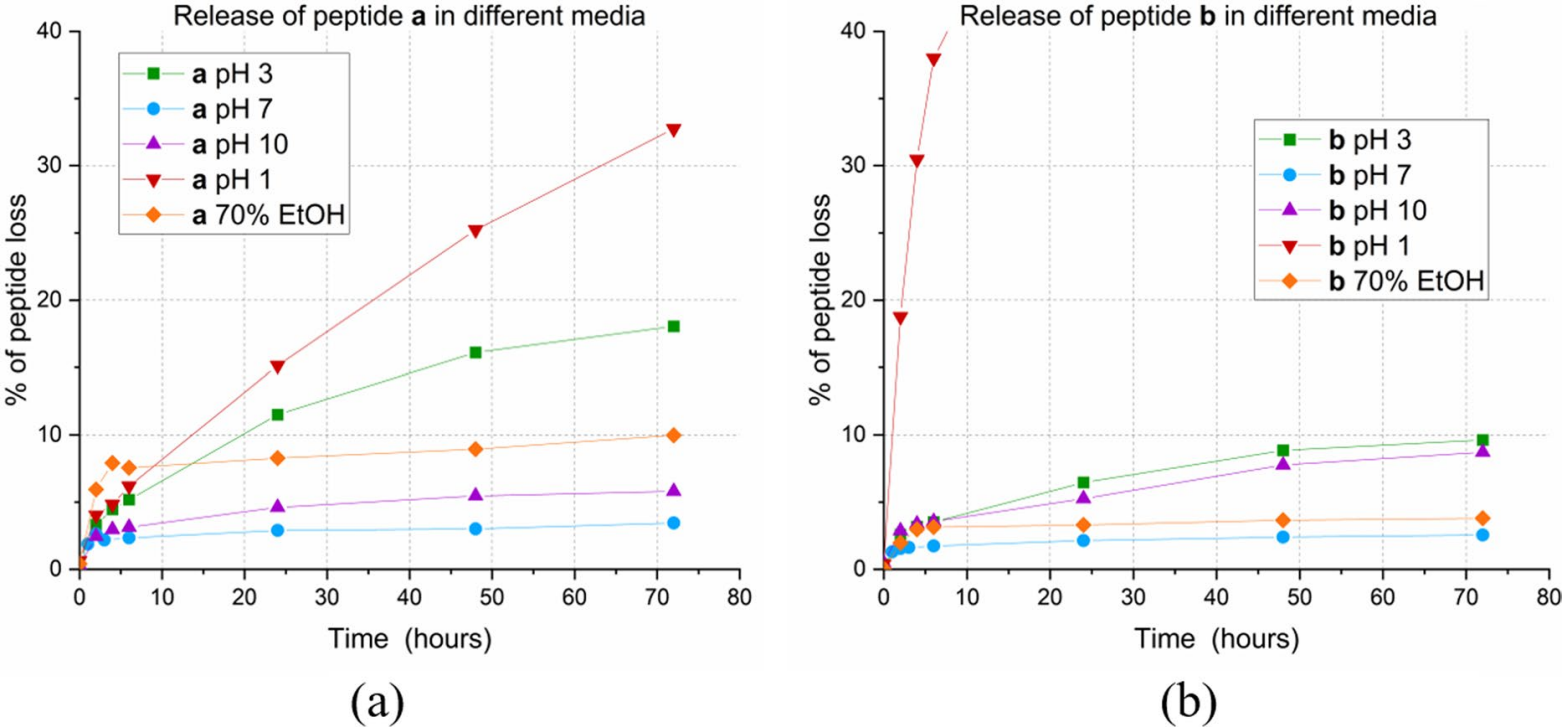
Percentage of peptide loss over time. **a** for H-Cys-Gly-Trp-Lys-NH_2_ from **r4**; **b** for H-Aox-Gly-Trp-Lys-NH_2_ from **r8**. The graph does not show data over 40% of peptide loss due to the chosen upper threshold in absorbance

In this study, the stability of the peptide-cotton conjugates was tested in different media, in acid, neutral, and basic aqueous solutions. In particular, cotton pieces from reaction **r4** (peptide **a**) and reaction **r8** (peptide **b**) were added to a citrate buffer (pH 3), a 3 M HCl solution, a PBS buffer (pH 7), a carbonate buffer (pH 10) and a 70%_v/v_ EtOH solution. The latter alcohol solution was used to test the stability of conjugates in sterilization treatments. Such treatments become necessary when linking to cotton a biologically active peptide whose activity is to be tested. The absorbance of the solution was monitored at 280 nm for three days, and in all solvents tested, some peptide release could be observed, as measured by increasing the absorbance of Trp. The absorbance of the solution was monitored at 280 nm for three days, and in all solvents tested some peptide release could be observed, measured by the increasing absorbance of Trp. In neutral solution, such as that of PBS buffer, peptide release in solution can be considered negligible being less than 5%. Strong acidic solutions, in contrast, are those in which peptide release was quite significant (Fig. 7).

The highest peptide loss was observed in 3 M HCl (pH 1) for both peptide **a** and peptide **b**. After three days, the release of peptide **a**, bound via thiazolidine, was 33%, while that of peptide **b**, bound via oxime, was much higher (Table 4). In the neutral PBS buffer solution, the loss of peptide is negligible with both types of bonds, which can therefore be considered rather stable. This is especially important when peptides with biological properties, such as antibacterial and antimicrobial peptides, are conjugated to cotton. The action of such conjugates will have to be ensured under physiological conditions that are well mimicked by PBS buffer at pH 7. Under such conditions, the peptide release after three days is of the order of 4% for the thiazolidine bond and even less than 3% for the oxime bond. For this reason, we can consider the two bonds to be extremely stable under physiological conditions.

**Table 4:** Percentages of peptide loss after 3 days in different media

	Peptide** a** (from reaction **r4**) *thiazolidine bond*	Peptide** b** (from reaction **r8**) *oxime bond*
pH 3	18%	10%
pH 7	< 4%*	< 3%*
pH 10	6%	9%
pH 1	33%	> 40%*
70% EtOH	10%	4%

*The lower and upper thresholds for the validity of the Lambert–Beer law were set at 0.05 and 1 in absorbance, respectively

In the basic solution, obtained with a carbonate buffer at pH 10, peptide release after the three days was observed to be around 6% for the thiazolidine bond and around 9% for the oxime bond, making it the more labile of the two.

Under slightly acidic conditions, given by a citrate buffer at pH 3. In contrast, the oxime bond is slightly more stable, with a 10% peptide loss, when compared to the 18% loss of the thiazolidine bond.

## Conclusions

In recent years, the need to create new textiles for use as the first protection in pathogen prevention has grown significantly. In this regard, the modification of textile materials, particularly with peptides, has become of great interest to the scientific community. Our work presents a study on the possibility of modifying cotton textiles with peptides using thiazolidine and oxime chemoselective ligations.

Cotton textile materials with different weaving were modified, first by oxidation (to obtain aldehyde moieties on cotton) and then by ligation of two model peptides by chemoselective ligation reactions. For this purpose, an enzymatic oxidation of cellulose in heterogeneous phase (insoluble cotton and soluble laccase/time solution) was successfully undertaken and the possibility of reuse of the oxidation solution for multiple times was demonstrated. This is very important for a possible industrial application of cotton modification. In fact, this allows water to be used as the only solvent and the enzymatic laccase solution to be reused to limit material waste and environmental pollution. Further work is undergoing in order to optimize the amount of laccase and TEMPO in the oxidation reaction.

The chemoselective conjugation reactions were then optimized to bind the peptide to cotton, and the best conditions consisted of a one-day reaction with at least 1 mM concentration of the peptide solution. The ligation of peptides to cotton was detected by colorimetric assays and demonstrated by FTIR and XPS analyses. Peptide loading was found to be time-dependent and can be modulated to the desired value. At any time, the reaction can be stopped by simply removing the cotton from solution, and loading can be verified at any time by UV analysis of the peptide remaining in solution.

The method here presented is applied to a model peptide with no biological activity. In the case of an active peptide, the degree of functionalization must be specifically evaluated by comparing the activity of the peptide in solution and when bound to cotton. Nevertheless, our method may serve as a general way for monitoring cotton functionalization reactions.

Both thiazolidine and oxime bonds are formed in a pH 5 buffer solution, thus demonstrating that this conjugation strategy also represents an environmentally friendly and sustainable step for cotton functionalization. A detailed comparison between thiazolidine and oxime bonds was carried out. In terms of ligation formation, both thiazolidine and oxime, under similar reaction conditions, last about 1 day and give comparable peptide loading in the order of magnitude of 1 mmol per gram of cotton. However, in the case of thiazolidine, for an efficient reaction, a reducing agent (TCEP) is required to prevent dimerization of the Cys-modified attacking peptide, especially when concentrated peptide solutions are required for the reaction. Thus, when possible, between the two chemoselective reactions, oxime seems to be a better choice.

The stability of the two newly formed bonds was also tested as a function of pH and solvent. The two bonds are stable to some extent in the pH range of 3 to 10. With minor differences, a slight release of the peptide from cotton was observed only after three days. A major release of the peptide is observed only in strongly acidic solutions. These small differences are important to know in the perspective of possible applications of cotton-peptide conjugates. Depending on the property desired to be imparted to the cotton surface, thiazolidine or oxime ligations may be chosen as the type of covalent bond. For some materials, a strong covalent bond may be best; for other applications, which require slow release of an active peptide, a slightly more labile bond may be preferable. In this case, thiazolidine or oxime can be chosen depending on the pH conditions of the environment in which the material will be used.

In conclusion, the study presented here suggests that chemoselective ligation strategies applied to cotton are very promising as a technique to functionalize cotton with peptides with an all-green procedure that also offers the possibility of modulating the stability of the final bond.

## Supplementary Information

Below is the link to the electronic supplementary material.

Supplementary file1 (DOCX 624 KB)

## Abbreviations

Cys: Cysteine
Aox: Aminooxyacetic acid
Lys: Lysine
Gly: Glycine
Trp: Tryptophan
TEMPO: 2,2,6,6-Tetramethylpiperidine-1-oxyl
TCEP: Tris(2-carboxyethyl)phosphine
DMF: Dimethylformamide
TFA: Trifluoroacetic acid
Boc: Tert-butyloxycarbonyl
Fmoc: 9-Fluorenylmethoxycarbonyl
DIC: N,N′-Diisopropylcarbodiimide
Oxyma: Ethyl 2-cyano-2-(hydroxyimino) acetate
DODT: 3,6-Dioxa-1,8-octanedithiol
