# Supplementary material for: Oxime and thiazolidine chemoselective ligation reactions: a green method for cotton functionalization
Source: Cellulose (Lond). 2023 May 18;30(9):5573–87. doi: 10.1007/s10570-023-05253-1 (PMC10193351; doi:10.1007/s10570-023-05253-1)
Supplement: Supplementary file 1 — Supplementary file1 (DOCX 624 KB) [file 10570_2023_5253_MOESM1_ESM.docx]

Oxime and thiazolidine chemoselective ligation reactions: a green method for cotton functionalization

Francesca Albini^1^, Barbara Biondi^2^ , Luana Lastella^1^, and Cristina Peggion^1*^

^1^ Department of Chemistry, University of Padova, 35131 Padova, Italy
^2^ ICB CNR, Padova Unit, Department of Chemistry, University of Padova, 35131 Padova, Italy

Supplementary Information

**Figure S1.** ^1^H NMR one-dimensional spectrum of H-Cys-Gly-Trp-Lys-NH_2_ (peptide **a**) (400 MHz, DMSO-d6, 298K).

**Figure S2.** ^1^H NMR one-dimensional spectrum of H-Aox-Gly-Trp-Lys-NH_2_ (peptide **b**) (400 MHz, DMSO-d6, 298K).


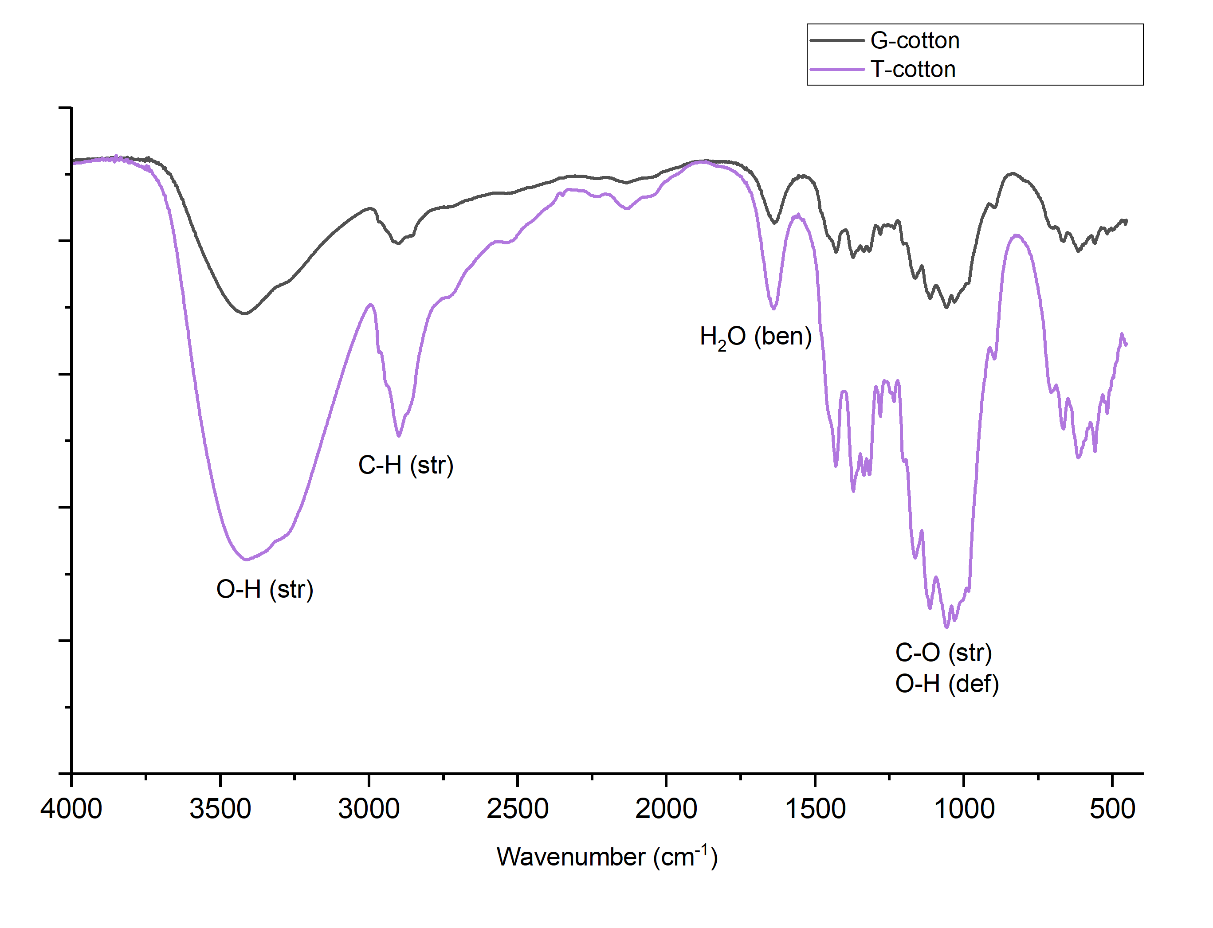


**Figure S3.** FT-IR spectra of G and T-cotton


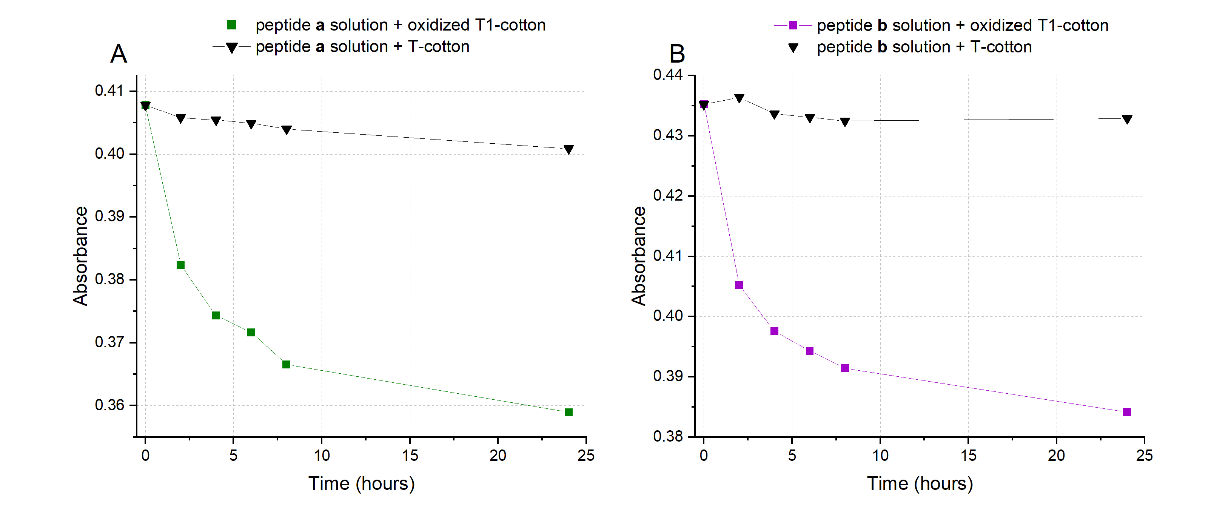


**Figure S4.** Absorbance at 280nm over time of (a) peptide **a** solution in reaction **r11**(b) peptide **b** solution in reaction **r12.** In black is shown the absorbance of the peptide solution in the presence of unoxidized T-cotton, in green or purple in the presence of oxidized T1-cotton.

**XPS data**

***Oxidized cotton (T1)***

| Name | Peak BE | FWHM eV | Area (P) CPS.eV | Atomic % | Q |
| --- | --- | --- | --- | --- | --- |
| O1s | 533.08 | 4.98 | 17238.36 | 80.42 | 1 |
| C1s | 287.00 | 0.79 | 1714.45 | 19.34 | 1 |

**Common Acquisition Parameters Table**

| ***Parameter*** |  |
| --- | --- |
| Total acquisition time | 2 mins 15.0 secs |
| Number of Scans | 1 |
| Lens Mode | Standard |
| Analyser Mode | CAE : Pass Energy 200.0 eV |
| Energy Step Size | 1.000 eV |
| Number of Energy Steps | 1351 |

***Cotton functionalized with peptide a from reaction r4***

| Name | Peak BE | FWHM eV | Area (P) CPS.eV | Atomic % | Q |
| --- | --- | --- | --- | --- | --- |
| N1s | 398.42 | 2.72 | 22281.87 | 2.72 | 1 |
| O1s | 531.37 | 4.24 | 420763.24 | 32.95 | 1 |
| C1s | 284.69 | 4.93 | 339684.78 | 64.33 | 1 |

**Common Acquisition Parameters Table**

| ***Parameter*** |  |
| --- | --- |
| Total acquisition time | 2 mins 15.0 secs |
| Number of Scans | 1 |
| Lens Mode | Standard Electrostatic |
| Analyser Mode | CAE : Pass Energy 200.0 eV |
| Energy Step Size | 1.000 eV |
| Number of Energy Steps | 1351 |


**Common Acquisition Parameters Table**

| ***Parameter*** |  |
| --- | --- |
| Total acquisition time | 27.1 secs |
| Number of Scans | 3 |
| Lens Mode | Standard Electrostatic |
| Analyser Mode | CAE : Pass Energy 200.0 eV |
| Energy Step Size | 0.100 eV |
| Number of Energy Steps | 181 |

***Cotton functionalized with peptide b from reaction r9***

| Name | Peak BE | FWHM eV | Area (P) CPS.eV | Atomic % | Q |
| --- | --- | --- | --- | --- | --- |
| O1s | 531.34 | 4.12 | 462016.75 | 33.82 | 1 |
| C1s | 284.68 | 4.74 | 360821.18 | 63.87 | 1 |
| N1s | 398.65 | 3.91 | 20199.50 | 2.30 | 1 |

**Common Acquisition Parameters Table**

| ***Parameter*** |  |
| --- | --- |
| Total acquisition time | 2 mins 15.0 secs |
| Number of Scans | 1 |
| Lens Mode | Standard Electrostatic |
| Analyser Mode | CAE : Pass Energy 200.0 eV |
| Energy Step Size | 1.000 eV |
| Number of Energy Steps | 1351 |


**Common Acquisition Parameters Table**

| ***Parameter*** |  |
| --- | --- |
| Total acquisition time | 27.1 secs |
| Number of Scans | 3 |
| Lens Mode | Standard Electrostatic |
| Analyser Mode | CAE : Pass Energy 200.0 eV |
| Energy Step Size | 0.100 eV |
| Number of Energy Steps | 181 |
